# Lymphangitic Pulmonary Metastases in Castrate-Resistant Prostate Adenocarcinoma

**DOI:** 10.1155/2012/980920

**Published:** 2012-08-08

**Authors:** Andrew Meyer, Rachel Angelica Mariani, Chadi Nabhan

**Affiliations:** ^1^Department of Medicine, Advocate Lutheran General Hospital, Park Ridge, Chicago, IL 60068, USA; ^2^Department of Pathology, University of Illinois at Chicago, Chicago, IL 60612, USA; ^3^Department of Medicine, Division of Hematology and Oncology, Advocate Lutheran General Hospital, Park Ridge, Chicago, IL 60068, USA

## Abstract

A 63-year-old man with castrate-resistant metastatic prostate adenocarcinoma with known osseous and pelvic nodal involvement presented with progressive dyspnea for one week. Complete cardiopulmonary evaluation revealed a restrictive lung defect that could not be attributed to any of his previous therapies. On presentation, physical examination revealed coarse breath sounds diffusely with hypoxemia. Computed tomography of the chest showed severe bilateral airspace opacities and ground-glass appearance most consistent with interstitial pneumonitis. The patient was intubated due to progressive hypoxemia and worsening respiratory status despite empiric antibiotics and high dose steroids. Subsequent emergent bronchoscopy with transbronchial biopsies revealed atypical intralymphatic cells that stained positively for prostate-specific antigen and prostatic-specific acid phosphatase, confirming the diagnosis of intralymphatic pulmonary metastasis from prostate adenocarcinoma. Lymphangitic pulmonary metastasis from prostate adenocarcinoma is exceedingly rare, with few reported cases that are biopsy-proven. Herein, we describe a rare case of biopsy-proven lymphangitic pulmonary metastasis in the setting of castrate-resistant prostate adenocarcinoma and provide a comprehensive literature review.

## 1. Case

A 63-year-old man who has metastatic prostate cancer with known osseous and pelvic nodal involvement presented with a one-week history of progressive dyspnea. The patient had presented originally with metastatic disease 5 years prior to admission. He was treated with complete androgen deprivation therapy (ADT) attaining biochemical complete response that lasted for 2 years. Subsequently, he received sipuleucel-T on a clinical trial demonstrating stable disease radiographically for 18 months. Radiographic and biochemical progression necessitated treatment with lenalidomide on a phase II clinical trial. In total, he received 4.5 months of lenalidomide achieving partial biochemical response and stable disease radiographically. However, lenalidomide was discontinued 3 months prior to presentation due to shortness of breath and fatigue attributed initially to the study drug. Pulmonary function testing at that time showed a restrictive pattern and cardiac evaluation was nonrevealing. Dyspnea improved slightly after stopping lenalidomide and further improved with pulse steroids. He subsequently received temsirolimus for 3 weeks before hospitalization with the above-mentioned complaints. 

**Figure 1:**
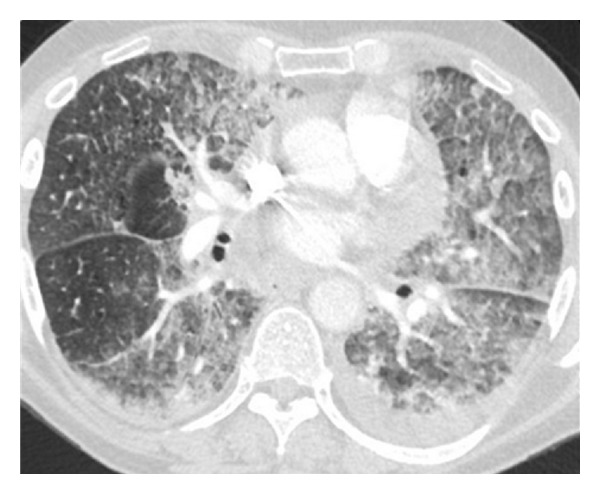
Computed tomography of the chest illustrating severe bilateral opacities with ground-glass appearance.

On presentation, physical examination revealed coarse breath sounds bilaterally with hypoxemia. Computed tomography (CT) of the chest showed severe bilateral airspace opacities and ground-glass appearance most consistent with interstitial pneumonitis (Figure 1).

**Figure 2:**
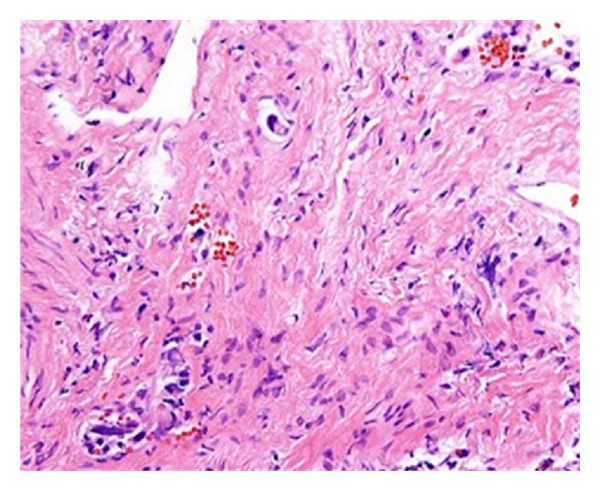
H&E, 40x. Atypical cells within a lymphatic lumen. The atypical cells have abundant cytoplasm and large nuclei with prominent nucleoli.

Empiric broad-spectrum antimicrobials and high-dose steroids were initiated but his respiratory status and hypoxemia worsened further. Emergent bronchoscopy revealed normal tracheobronchial trees bilaterally without evidence of inflammation or edema. Transbronchial biopsies revealed atypical intralymphatic cells with abundant cytoplasm and large nuclei with prominent nucleoli (Figure 2).

**Figure 3:**
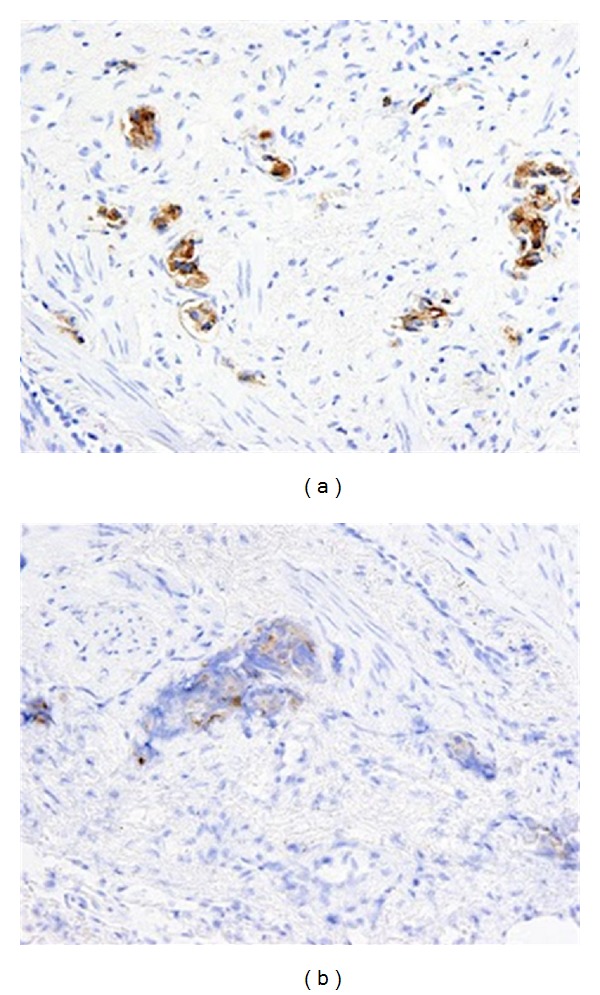
Prostate-specific antigen (PSA) and prostate-specific acid phosphatase (PSAP) stains, 40x. Metastatic prostate adenocarcinoma within lymphatic spaces, as demonstrated by PSA and PSAP positivity.

Subsequent immunostains for prostate-specific antigen (PSA) and prostatic-specific acid phosphatase (PSAP) confirmed intralymphatic involvement by prostate adenocarcinoma (Figures 3(a) and 3(b)). While systemic chemotherapy was offered in an attempt to improve his respiratory condition, the family opted for palliative measures, and the patient died 24 hours after extubation.

## 2. Discussion

Pulmonary lymphangitic carcinomatosis is pathologically described as the presence of tumor thrombi in the lymphatic vessels of bronchovascular bundles, interlobular septa, and pleura [1]. Clinically, the disease process can manifest as progressive dyspnea with subacute cor pulmonale and portends a poor prognosis. Imaging studies characteristically show multiple linear densities forming a reticular network with thickened and irregular bronchovascular bundles. Another common radiographic presentation of pulmonary lymphangitic carcinomatosis is the “tree-in-bud” pattern, which describes bronchiolar luminal impaction that outlines the normally invisible peripheral airway branching. Diagnosis is usually made based on clinical grounds but can be definitively established by obtaining a transbronchial lung biopsy.

In prostate cancer, lymphangitic pulmonary involvement is exceptionally rare, occurring in less than 0.2% of patients [9]. Nodular involvement, however, is more commonly observed. A retrospective review of preoperative chest X-rays for 91 patients with advanced prostate cancer undergoing bilateral orchiectomy revealed 3 patients with bilateral coarse infiltrates consistent with lymphangitic spread [10]. Several cases of presumed pulmonary lymphangitic spread from prostate cancer without confirmatory lung biopsy were identified [5, 11, 12]. Pulmonary lymphangitic spread of prostate cancer that is confirmed pathologically via lung biopsy is rarely reported. To our knowledge, only 7 cases described biopsy-proven lymphangitic metastasis from prostate cancer, but surprisingly none occurred in the castration-resistant setting (Table 1) [2–8]. We describe a rare case of biopsy-proven pulmonary lymphangitic metastasis in the castration-resistant setting. 

## Figures and Tables

**Table 1:** Previously reported cases of lymphangitic pulmonary metastasis of prostate cancer.

Reference	Status of PC at time of lymphangitic spread	Initial pulmonary presentation	Lung biopsy	Outcome
Miseria et al. [2]	Hormone sensitive	Diffuse interstitial infiltrate with reticulonodular pattern	Done	Clearing of infiltrates with ADT
Rossi et al. [3]	Hormone sensitive	Bilateral multiple small nodules	Done	Given ADT, outcome not reported
K. S. Miller and J. M. Miller [4]	Hormone sensitive	Diffuse, bilateral, reticulonodular infiltrate	Done	Not reported
Cohen et al. [5]	Hormone sensitive	Bilateral interstitial infiltrates	Done	Received ADT followed by chemotherapy with radiographic improvement
Heffner et al. [6]	After failing first-line hormonal therapy with DES	Large bilateral effusions with interstitial infiltrate	Done	Second-line hormonal therapy given
Arriero et al. [7]	Hormone sensitive	Bilateral interstitial densities with perihilar predominance	Done	Improvement with ADT but suffered SCD of unknown cause 4 months later
Schwarz et al. [8]	Developed after failing first-line therapy	Diffuse infiltrations and nodularity	Done	Improvement after orchiectomy

ADT: androgen deprivation therapy, DES: diethylstilbestrol, SCD: sudden cardiac death.
